# Data-driven approaches can harness crop diversity to address heterogeneous needs for breeding products

**DOI:** 10.1073/pnas.2205771120

**Published:** 2023-03-27

**Authors:** Jacob van Etten, Kauê de Sousa, Jill E. Cairns, Matteo Dell’Acqua, Carlo Fadda, David Guereña, Joost van Heerwaarden, Teshale Assefa, Rhys Manners, Anna Müller, Mario Enrico Pè, Vivian Polar, Julian Ramirez-Villegas, Svein Øivind Solberg, Béla Teeken, Hale Ann Tufan

**Affiliations:** ^a^Digital Inclusion, Bioversity International, 34397 Montpellier, France; ^b^Department of Agricultural Sciences, Inland Norway University of Applied Sciences, 2318 Hamar, Norway; ^c^International Maize and Wheat Improvement Centre, Harare, Zimbabwe; ^d^Center of Plant Sciences, Scuola Superiore Sant’Anna, 56127 Pisa, Italy; ^e^Biodiversity for Food and Agriculture, Bioversity International, 00100 Nairobi, Kenya; ^f^Digital Inclusion, International Center for Tropical Agriculture, Arusha, Tanzania; ^g^Department of Plant Sciences, Wageningen University and Research, 6708PE Wageningen, Netherlands; ^h^Crops for Nutrition and Health, International Center for Tropical Agriculture, Arusha, Tanzania; ^i^International Institute of Tropical Agriculture, Kigali, Rwanda; ^j^International Potato Center, 15023 Lima, Peru; ^k^Climate Action, International Center for Tropical Agriculture, 763537 Cali, Colombia; ^l^International Institute of Tropical Agriculture, 200001 Ibadan, Nigeria; ^m^College of Agriculture and Life Sciences, Cornell University, 14853 Ithaca, NY

**Keywords:** genebanks, plant breeding, gender, genotype by environment interactions, socioeconomic heterogeneity

## Abstract

This perspective describes the opportunities and challenges of data-driven approaches for crop diversity management (genebanks and breeding) in the context of agricultural research for sustainable development in the Global South. Data-driven approaches build on larger volumes of data and flexible analyses that link different datasets across domains and disciplines. This can lead to more information-rich management of crop diversity, which can address the complex interactions between crop diversity, production environments, and socioeconomic heterogeneity and help to deliver more suitable portfolios of crop diversity to users with highly diverse demands. We describe recent efforts that illustrate the potential of data-driven approaches for crop diversity management. A continued investment in this area should fill remaining gaps and seize opportunities, including i) supporting genebanks to play a more active role in linking with farmers using data-driven approaches; ii) designing low-cost, appropriate technologies for phenotyping; iii) generating more and better gender and socioeconomic data; iv) designing information products to facilitate decision-making; and v) building more capacity in data science. Broad, well-coordinated policies and investments are needed to avoid fragmentation of such capacities and achieve coherence between domains and disciplines so that crop diversity management systems can become more effective in delivering benefits to farmers, consumers, and other users of crop diversity.

The use of large datasets and computational methods has transformed the practice of crop breeding. This is driven by i) the lowering costs per data point of genomic and high-throughput phenotyping data, ii) speeding up breeding cycles, and iii) using computationally intensive data analytics. This is leading to a change in strategy and resource allocation that underpin crop breeding (1–3). Data-intensive approaches have led to a wider change in scientific practice, in which research makes increasingly intensive use of data, and hence becomes more driven by data than by hypotheses (4). The availability of larger data volumes is provoking a change where before sparse data were interpreted with the aid of convenient simplifying assumptions. For example, genomic data unveiled the complexity of the genetic basis of quantitative traits, showing the limits of additive models (5). Data also redraw disciplinary boundaries and collaborations as they become the common currency of interdisciplinary integration (6). Data-intensive approaches have not only become important in genomics but also in other research disciplines, including environmental characterization (7), socioeconomic characterization (8), trait prioritization (9), on-farm variety testing (10), and other applications. Data-intensive approaches use an increased volume, speed, and a broader range of data within certain domains. We define data-driven approaches as taking the next step by integrating different types of data using relatively flexible, inductive methods. They contrast with model-driven approaches, which start from a higher level of conceptual coherence and causal understanding. Models have a specific role to play in knowledge integration (for example, crop growth simulation), but we believe that as an overall strategy, data-driven approaches are more likely to be successful in bringing together different disciplines (11).

This perspective reflects on the changes brought by data-driven approaches in the context of crop diversity management for sustainable development in the Global South. “Global South” refers here to low- and middle-income countries in Latin America, Asia, Africa, and Oceania; high-income countries on other continents will be referred to as “Global North.” Our reflections should be relevant to researchers, policy makers, and investors working in or for the Global South. “Crop diversity management” encompasses the work on crop genetic innovation, conservation, and use covered by genebanks, breeding programs, as well as farmers, and others. We see this as a single field of work (12) and expect that boundaries between conservation and innovation may be further blurred by data-driven approaches, as we detail below. Our focus on sustainable development is embedded in international agricultural research. The authors are associated with CGIAR as staff or long-term collaborators.

In this context, more intensive use of data produces important new possibilities for more effective crop diversity management. First, an important opportunity is that data-driven strategies make it possible to connect different sources and types of data to gain insights into complex systems. This is relevant for crop diversity research, which needs to accommodate the complexity of cropping systems and rural livelihoods and the multiple policy demands placed on agricultural research and development. This includes demands for including food security, safety and quality, ecosystem services, climate resilience, and social justice. Data-driven approaches can respond to the call for “systems-based breeding,” which should respond to the complex demands placed on agriculture and to assess trade-offs between the different values involved (13).

The second way in which data-driven approaches have an impact on crop diversity use in breeding is through the ability to make productive use of data that are more “noisy” (high variance) but at the same time more representative of real crop diversity use conditions. These data include large datasets from user preference studies and on-farm testing networks. Such large-but-noisy datasets were previously unavailable, and the high cost of crop trials has usually meant that smaller but more precise datasets were favored to estimate heritability and breeding value of crop traits. However, digital media make it less costly to collect data across wider areas, for example, directly involving farmers and other crop diversity users in data collection through crowdsourced citizen science strategies, which we discuss below. These new approaches are generally less precise and afford less experimental control (for example, holding crop management constant in farmer-led trials), but they make two important complementary contributions. First, these methods gain in terms of the representativeness of crop use environments so that experiments and observations resemble more closely how farmers and others use crop diversity in real life. Second, these new approaches are better able to address the complexity of the studied system, involving not only plant biology but also the environmental and human aspects of crop diversity use. In Fig. 1, we map several data-generating approaches in crop diversity research along these two dimensions, representativeness and complexity.

**Fig. 1.**
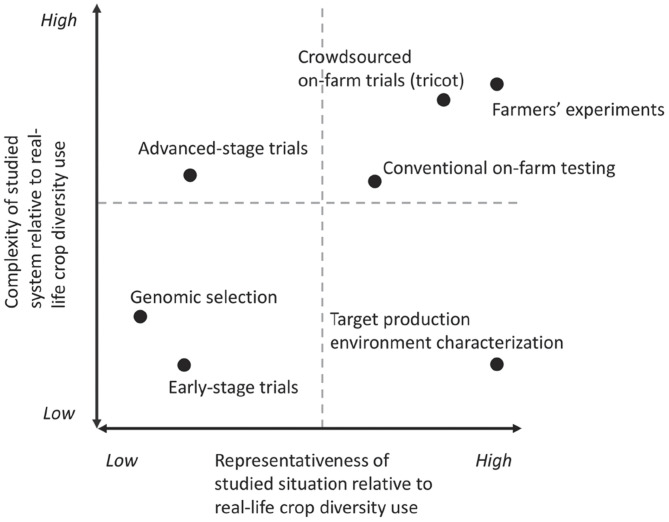
Data-driven integration. Different methods differ in the degree to which they address the complexity of the crop use context (involving biological, environmental, and human aspects) and represent the diversity of the use contexts (environments, market segments, etc.). Different data-driven approaches can bridge different areas of the diagram by linking different datasets to provide insights into the heterogeneous needs for traits and breeding products. Data-driven integration links datasets through integrative analytics. Combining different datasets in a single multiway analysis can address complexity and ensure representativeness. Different methods are placed to show relative positions, based on the authors’ judgment.

Together, the ability to handle higher complexity and achieve greater representativeness increases agricultural R&D capacity to generate a portfolio of research products that demonstrate adaptability to their use contexts and deal effectively with the different trade-offs between the different demands placed on crop production. The resulting portfolio will have a better joint fitness to respond to heterogeneous needs, including the demands of crop diversity users and policy goals for livelihoods and production systems. The emergence of data-driven approaches does not mean a fundamental shift in scientific epistemology in that we do not expect that they will replace approaches that are focused on high-precision investigation or single-factor studies (6). In breeding, high selection efficiency requires experimental control of nongenetic variation and a focus on a reduced set of traits. The question is how a configuration of different approaches, connected in a data-driven way, can simultaneously embrace selection efficiency, fitness, trade-offs, and adaptability. Any strategy to make effective use of crop diversity should accommodate and connect approaches spread across the quadrants in Fig. 1 (for an example, Fig. 2). In this perspective paper, we discuss some of the methodologies cited in this diagram to trace how new data-driven approaches can support better use of crop diversity.

**Fig. 2.**
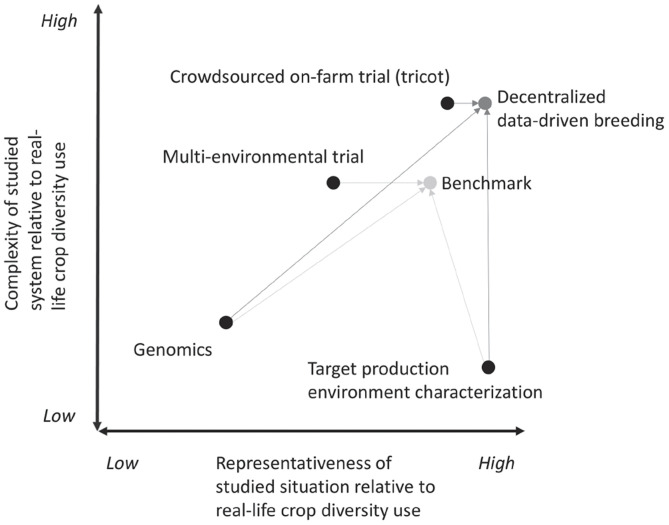
An example of data-driven integration, based on ref. 58. Two approaches are being compared: decentralized data-driven (3D) breeding and a benchmark. The arrows indicate for two approaches how data are linked into a single analytical model. Each model seeks to maximize both complexity and representativeness of the information that guides plant selection. 3D breeding covers more complexity because tricot on-farm testing achieves better predictions of farmers’ overall appreciation of varieties (a complex “trait” involving many variables apart from yield). Also, it attains higher representativeness because more environments could be sampled (locations and planting dates). Target production environment (TPE) characterization increases representativeness in both cases. Biased sampling of environments (locations and planting dates) in the multienvironmental trial limit representativeness, however, which could not be entirely corrected by linking with TPE characterization data.

## Overcoming Historical Barriers

Data-driven approaches disrupt a series of practices that were foundational to modern breeding but implied various barriers to crop diversity use. The current breeding paradigm was formed by the revolution in inferential statistics initiated by William S. Gosset, Ronald Fisher, and others in the first half of the 20th century, and the “Green Revolution” breeding focus epitomized by Norman Borlaug. Three features of this approach are barriers for crop diversity use.

First, this approach focused on how selection affects mean responses and treats genotype-by-environment interactions (Gx-E) and nonadditive effects generally as nuisance factors (5, 14). Specifically, the focus on mean response and broad adaptation discouraged breeders from considering heterogeneous crop use contexts (2, 15). Second, the approach that emerged in the first half of the 20th century introduced replication and randomization into crop experimentation. This innovation enabled more precise estimates by accounting for statistical error. However, this made it more difficult for farmers to implement on-farm trials and drove a wedge between researchers’ and farmers’ experimentation practices (16, 17). Thirdly, public breeding in the Global South has historically focused mainly on productivity and stress tolerance. Other traits were generally included in selection strategies in a piecemeal way, in the absence of robust market intelligence (18). Together, these three barriers impeded more effective crop diversity use for adaptation to specific environments, addressing diverse farmers’ needs, or selection for a wider range of traits.

At present, breeding programs increasingly need to address changing policy agendas that emphasize commercial interests, environmental concerns, and participatory approaches (19). In this respect, public breeding is broadening the range of factors in the breeding product design process (18). Here, we suggest that breeding programs can best address these new demands through data-driven approaches. This requires not only institutional change to enable interdisciplinarity but also changes in methodological routines, which require careful attention.

## Better Use of Plant Genetic Resources

Crop genetic diversity in genebanks is fundamental for demand-driven breeding. Genebanks supply diversity of local crop populations, which have coevolved with local conditions and show potential for continued evolution, as well as genes to address emerging diseases and other challenges (20, 21). FAO WIEWS indicates that there are more than 5.7 million accessions stored in 831 genebanks around the world, which represents a large amount of diversity. However, this could be overestimated; as the majority of the accessions are not described, backlogs are common in regeneration and characterization of material, and duplication may occur (22). Economic research shows that genebanks have a very high benefit to cost ratio, but lacking or deficient accession data reduce the value of genebank collections, increase search costs, and reduce use of particular accessions (23). Furthermore, while international policy frameworks emphasize the need for coordination between ex situ and in situ conservation, actual examples of coordination are rare (24). For breeding programs, incorporating traits from farmer varieties and wild materials is already a significant investment in itself. A lack of information is an important additional barrier to more effective use of existing crop genetic resources in genebanks.

Data-driven solutions address some of the main challenges in generating value from genebank collections. Data-driven approaches have the potential to bridge genebank conservation, on the one hand, and use of diverse materials in representative, complex use contexts, on the other.

So far, much work has focused on adaptability of genebank accessions by linking accessions’ georeferenced collecting sites to agroclimatic data. Filtering on climatic adaptability, the Focused Identification of Germplasm Strategy (FIGS) narrows down the number of candidate accessions for a given area (25). Methodologically, FIGS has led to interesting innovation in analyzing multiway datasets, simultaneously addressing biological and spatiotemporal dimensions (26). While this approach can handle the representativeness dimension of Fig. 1, in that it uses the growing environment to predict traits, so far, it has focused on climate and adaptation traits and not the full complexity of target crop use contexts and traits. Direct use of genebank collections by farmers, farmer organizations, backyard gardeners, and citizen scientists in countries around the world has spurred new data-driven approaches. Some of this is part of deliberate strategies to enhance the use of plant genetic resources, especially in crops that have not benefited from long-term breeding programs. For example, from 2013 to 2017, the genebank of the World Vegetable Center has distributed over 42,000 seed kits containing over 183,000 vegetable seed samples to smallholder farmers in Tanzania, Kenya, and Uganda (27). Feedback data from farmers and consumers can generate important new insights into genebank samples.

This is the explicit goal of the Seeds for Needs initiative, in which over 40,000 farmers across Africa, Asia, and Latin America take part in testing and selecting germplasm adapted to their needs (28). Multiple benefits accrue from farmers and other citizens accessing diversity directly, both the collection of feedback data and a greater use of superior materials. In Ethiopia, it was shown that farmers were able to identify superior landraces of durum wheat, which were directly released as varieties (10, 29).

The resulting data are potentially “noisy” but are produced in real farming systems and involve a multifaceted perspective on crop diversity, beyond environmental adaptation or the search for a small set of traits. As a result of these initiatives, genebanks obtain performance information produced under highly diverse, but representative conditions, which can enhance the value of genebanks and the use of plant genetic resources. It is well known that characterized and sequenced accessions have a higher value than accessions with little information (23), but direct evaluation in farming systems can enhance this value further, linking genebank collections directly with the potential fitness of accessions in crop use contexts (Fig. 1).

To fully realize the potential of data-driven approaches that are more inclusive of complexity and aiming at representativeness, genebanks would need to reposition themselves. Right now, the strengths of genebanks are their collections, and they would need to develop stronger capacities in data management (31) and managing relationships with farmers, breeders, and other genebank material users, as part of a more “circular” model of crop diversity conservation and use (32). This means that policies and funding are needed for genebanks to broaden their role from being a provider of materials to a facilitator of the use of crop diversity.

## Insights from Systems Biology and Omics

After the initial enthusiasm about “big data” waned, it became increasingly clear that deductive approaches were ill fitted in unraveling the complex determinants of target organisms’ trait values. The omics technologies, which include genomics, proteomics, and metabolomics, can now trace molecular processes and their relation across the flow of information from DNA to proteins and back. The large-scale application of these approaches has demonstrated that quantitative traits have a sheer complexity that cannot be reduced to additive effects (33). This complexity emerges at the genetic level from locus–locus interactions (34), phenotypic plasticity (35), heterosis (36), and from the small effect size of most quantitative trait loci (QTL) (37). It also emerges at higher levels of biological organization, including genotype-by-environment interactions (GxE; next section).

The limitations of deductive approaches in breeding can be seen in the handful of applications of gene-based innovations, either using transgenesis or introgression. These success stories, although of extraordinary impact, are limited to oligogenic traits, which can be significantly altered by manipulating few genomic loci, as in the cases of submergence tolerance (38), disease resistance (39), or increased accumulation of metabolites (40).

The realization of this complexity fostered the use of agnostic genomic selection approaches in breeding that do not rely on functional interpretations of DNA variants but rather on modeling the effects of genome-wide allelic combinations (36). These data-driven approaches enable enlarging the agrobiodiversity basis that can be screened by breeding while furthering our understanding of trait determinants (41).

Individual traits targeted by breeding do not exist in a vacuum but rather are intimately correlated in a systems biology dimension. In the quest for selection efficiency, breeding programs cannot assume that selection for one trait will not affect any other aspects of plant performance. They need multitrait selection models to support genetic gain (42, 43) and tackle trait correlations and trade-offs (44) for effective crop improvement.

Data-driven methods can support genomics-assisted breeding by expanding the data available to train models. This enables deep learning approaches to improve genomic prediction models, capitalizing on early evidence that deep learning may better capture nonlinear patterns more efficiently than current models (45). A recent study shows that integration of data from different disciplines is possible in a data-driven framework, including genomics and climate modeling to generate recommendations for cross-border crop diversity conservation policies (46). Such multidisciplinary studies are made possible by the integration of heterogeneous datasets and the imaginative use of proxies. The flexibility brought about by the data revolution means that breeding can now leverage data sources and experimental designs that were not part of traditional improvement methods, including unbalanced designs (47, 48), genomic-based modeling of the evolutionary history of crop diversity (49), and farmers’ traditional knowledge associated with crop genetic resources (50). In a data-driven framework, partial and unbalanced datasets may be integrated deriving information that is superior to that resulting from the sum of the parts.

Data-driven methods also help in collecting better data on breeding target traits. Phenomics platforms can now routinely collect thousands of data points in systematic experiments both in controlled and field conditions, enabled by rapid technological advancements in imaging and computational technology (51, 52). In one day, a fixed-wing drone can generate high-resolution images for hundreds of hectares. With these data, breeders can discern subtle, yet meaningful, differences in phenotypic expression, increasing selection accuracy (53). However, drones face legal restrictions in many countries in the Global South. Another limitation is that drones observe crops from top-down viewpoints and therefore miss important crop traits and responses. For example, many fungal diseases first progress from the bottom stems and leaves, which is not easily visible from above. Rovers (ground-based vehicles) can provide proximal sensing imagery with several high-resolution cameras at different angles (54). Rovers take more time per unit of land than drones and have yet to reach public breeding programs in the Global South, which often struggle with poor internet connectivity and inconsistent power supply. While both drones and rovers have an important role to play, in the foreseeable future, their use in public breeding in the Global South will be limited.

To address the need for affordable imaging, mobile phone-based platforms could provide several options (55). A few private and public organizations have successfully developed smartphone-based image recognition systems to detect and diagnose foliar pests, diseases, and mineral nutrient deficiencies (56). Currently, a low-cost, smartphone-deployable imaging system is being designed for phenotyping in breeding programs in the Global South (Project Artemis). Such a low-cost smartphone-based phenotyping tool could help to bridge the gap between on-station and on-farm varietal evaluation.

Data-driven approaches also allow breeders to tap into farmers’ and consumers’ perceptions of quality and desirability to support varietal development. For example, sensory testing in the tomato has been adapted to deal with larger sets of crosses and to link flavor to the underlying chemical interactions (57). Data-driven decentralized breeding (3D breeding) can scale up varietal testing in larger sets of environments. Furthermore, by targeting farmers’ evaluations, 3D breeding can significantly increase genetic gain (58) and contribute to closing the gap between expected and realized gains in improved crop technologies in smallholder farming (59). (We discuss environmental adaptation in the next section.)

Following an inductive, data-driven approach that focuses on complex interactions, breeding can deal with trade-offs in improving a larger set of complex traits, while maintaining diverse breeding populations. Optimal contribution selection can combine production traits with quality and use traits, guiding genomic selection toward the establishment of multiple allele pools targeting local needs (60). The concurrent development of models more capable of characterizing and predicting Gx-E under varying conditions may further support selection pipelines (61). Selection intensity must be balanced with diversity to fuel incremental improvement for a number of target environments. Prebreeding tools and multiparental segregating populations are being developed to focus multidisciplinary research, avoid over-simplification, and embrace the diversity of trait determinants and trade-offs (62).

These data-driven approaches contribute to a new phase in the coevolution between people and plants (63). It may seem that the highly technical approaches increase the distance between people and plants, but they can bring us closer if they connect plant selection with fitness for human use, across the dimensions indicated in Fig. 1.

## Adaptation to Environment and Management

Matching crop traits and varieties to diverse growing conditions remains a challenge in managing crop diversity. Plant selection is often done in selection environments with near-optimal conditions to ensure heritability or under “managed stress” to select stress-tolerant genotypes. However, crop performance in such environments can have a low correlation with performance in target environments (64). Breeders strove to reduce GxE by selecting for stable genotypes with low interactions (broad adaptation) (2, 15) or favored varieties with stronger GxE but superior performance in high-input environments (65). Breeders were seldom able to disentangle the causality behind GxE. They had to deal with several limitations: absent or limited complementary environmental and crop physiological data, small trial sizes, and statistical methods which generally treated interactions as either a nuisance factor or as a basis for broad classification of trial locations. This limited the effectiveness of breeding as well as location-specific variety recommendations.

These challenges can limit the translation of research findings on crop diversity to use applications (66). For example, the identification of drought tolerance genes is determined by recording plant survival to artificially induced stress, but this is often difficult to translate to performance under realistic conditions in which there are generally trade-offs between the ability to survive drought stress and overall productivity (67).

A closely related challenge is that breeding is addressing a moving target, as production environments and crop management are both subject to accelerated change due to climate change, environmental degradation, and strategies to respond to these challenges. For example, conservation agriculture, a management strategy that involves reduced soil tillage provides a new challenge to breeding as it requires crops with plastic root phenotypes that can avoid the harder bulk soil but take advantage of more frequent biopores (68). A common response to the challenge of environmental variation has been to argue that broad adaptation addresses this issue. However, breeding for broad adaptation is likely to be suboptimal, as ecological theory predicts higher productivity for a larger set of more narrowly adapted varieties (69).

Subdividing breeding environments can help to address some of the challenges posed by GxE. For example, mega-environments have been used to target the efforts of CIMMYT’s global breeding programs (70). While such an environmental subdivision addresses large-scale, average environmental conditions, it does not in itself address temporal variation or variation at smaller spatial scales or in crop management.

Data-driven strategies provide a possible solution by linking trial performance data directly with data on the environmental conditions occurring in those trials (58). The recent availability of daily weather data for large parts of the globe facilitates the use of environmental covariates in trial data analysis and crop modeling to address the challenge of representativeness, while monitoring biological complexity at the same time (71). Analytically, this has been made possible by new statistical methods, which were not available to previous generations of researchers (72, 73).

The use of environmental covariates in trial analysis is still not a routine practice in many breeding programs but has led already to important insights (74). Going to the maximum in this direction, “envirotyping” has been coined to describe a strategy in which environmental data are collected at the lowest experimental level possible, including data on weather, soil, crop management, and companion organisms (7). To make better use of available data and to address complexity, data-driven approaches can go beyond additive models and use mechanistic crop modeling to simulate biological processes. Crop models serve to assess genotypic adaptability beyond trial conditions by simulating crop performance for different temporal and spatial extents. This can help to translate findings to target environments, assess trade-offs between different traits under production conditions, and predict how much they contribute to the overall breeding value (75–77). Directly linking crop models to genomic analysis makes it possible to link two levels of biological complexity in more direct ways, which offers significant potential for improving breeding efficiency (78).

An example of crop modeling that has led to relevant new insights in breeding is a study of upland rice in Brazil (79). This study shows the relative importance of different environmental stresses under future climates and concludes that breeding should move away from a focus on broad adaptation and carefully place crop trials geographically while devoting specific attention to stress tolerance traits in different environments. Crop modeling addresses a dimension of representativeness that crop trials cannot address directly (future conditions) and provides more detailed insights into the causal pathway from environmental conditions to ecophysiological mechanisms. Involving crop modeling directly in trial data analysis can increase efficiencies, for example, by focusing selection on highly heritable physiological proxy traits that contribute to yield (76). This shows that crop modeling can inform about GxE and the need for diverse responses back into plant selection for higher efficiency, bridging between trials that emphasize representativeness or experimental control.

On-farm trials are an important way to elucidate GxE interactions under representative crop management conditions. New data-intensive research has linked on-farm trials to environmental covariates using triadic comparisons of technology options (tricot) approach (80), in which farmers generate data by ranking the performance of small incomplete blocks. This approach has shown the differential response of Central American common bean to heat stress, Ethiopian durum wheat to cold stress, and Indian bread wheat to rainfall and radiation patterns (10). This work addresses both dimensions in Fig. 1. It takes into account the multidimensional overall evaluation of farmers (addressing complexity) and takes place in real production environments (addressing representativeness). Going one step further, the Ethiopian on-farm data have been linked to genomic data, showing that genomic selection can be greatly enhanced by on-farm and environmental data (58). This last work links high representativeness with high complexity but also enables translation to low complexity in environments with more homogeneous crop management to increase selection efficiency (Fig. 2). It is therefore a groundbreaking example of the potential of data-driven approaches to combine improved selection efficiency with a focus on fitness in the target environment. A next step in this area of work is to assess the feasibility of systematically implementing on-farm genomic selection in breeding programs, deploying optimized training populations directly on farms.

More methodological research is needed to make further progress in this area. Data analysis generally involves tools that are relatively new and sometimes immature, that are not routinely used by breeding programs and that are not usually used in combination with each other. To gain GxE insights from trials, data covering a range of environmental conditions are needed. Data synthesis can combine datasets from different origins to overcome data paucity (67, 81). To make this possible, barriers to data exchange need to be removed (moving toward open data), incentives for data sharing need to exist (for example, citation of datasets), data synthesis methods need to be developed and refined, and data standardization should be in place as much as possible.

Most analyses focus on the interaction between environments and yield but ignore interactions with management. The importance of such GxExM interactions is increasingly recognized (75), but empirical on-farm studies are still rare. Also, there may be GxE for traits other than yield such as product quality or its marketability that are best evaluated by farmers or consumers (82). More generally, it can be argued that the complexity of cropping systems, livelihoods, and markets is rarely addressed directly in the analysis of breeding trials. Large-scale farmer evaluations of varieties allow this type of complexity to be studied, but new analytical approaches are needed to extract relevant knowledge from the resulting information.

## Demand for Diversity: Gender and Social Differences

Variety traits have relevance throughout the different steps of crop production, processing, marketing, and consumption. The performance and user perception of new varieties during each of these steps will influence the perception of value and adoption of new varieties. The principles of demand-led breeding put the perceptions of these end users at the forefront of varietal design (83). At the same time, there is an increasing body of evidence documenting how gender and social differences within each of these user groups shape trait preferences and varietal adoption (84, 85). Breeding product design choices therefore affect who may use and benefit from new breeding products (86).

It is clear that breeding programs need to understand gender and social differences as drivers of trait preferences to achieve adoption, but they need to overcome a major barrier to achieve this: the dearth of relevant socioeconomic data. Gender data are notoriously sparse in development. This data dearth is even more pronounced when considering qualitative data, where datasets are seldom systematically tagged with identifiable keywords, making them unfindable in practice (87).

Lack of data limits studies on gender and social differences in crop improvement as well and is due to several issues. The first issue is that simply data are not sex-disaggregated. A recent study (85) shows that less than half of the studies disaggregated the adoption of climate-adapted varieties by sex. One important reason for this is that studies on crop trait preferences or adoption take the household as their unit of analysis, rather than the individual decision-maker (88). When women are included only as household heads, gender differences are attributed to different household structures, and data from women living in male-headed households are rendered invisible (89). This means that studies can only differentiate between male-headed and female-headed households. Usually, female-headed households are a small proportion of total households (90). A study that did differentiate between men and women as individual decision-makers found that maize adoption patterns were different between male–headed households, female-headed households, and women in male-headed households (91).

Another issue is the availability of data on gender in combination with other data on socioeconomic characteristics and social identity. These data are necessary to understand intersectionality, which concerns how different social aspects beyond gender (class, race/ethnicity, and others) intersect in shaping exclusion and inclusion (92). Moving away from homogeneous comparisons of men and women, it becomes important to integrate social identities and household characteristics that may interact with gender to shape trait preferences to determine the success of new varieties (9).

A third issue is the need for analytical approaches to derive insights from gender and socioeconomic data and inform decision-making. Decision-makers will need insights into the costs and (social) benefits of different strategies to address gender and socioeconomic differentiation in crop diversity management.

Data-driven approaches can address this challenge at various points in crop improvement: the generation of product profiles, evaluation of varieties and variety-specific products with users, and the tracing of variety losses, demand, and adoption. A recent study in Nigeria linked user trait prioritization experiments to socioeconomic data and showed that gender interacts with poverty, food security, and other factors to shape priorities (9). The study linked trait preferences related to the production of gari, a food product obtained from processing cassava, to detailed socioeconomic data on each individual user (8, 93). Results showed that quality traits were more important for members from food insecure households, and gender differences between men and women increased among the food insecure where women prioritize quality traits more. Respondents from poor and nonpoor households prioritized traits equally, but poor women prioritized quality traits more. Also, insights were gained into intrahousehold dynamics. Further integration of such approaches with other data-driven approaches e.g., tricot (80) will make it possible to track the effect of these differential preferences on on-farm selection and adoption choices.

A first limitation of these strategies is that they are often limited to a set of traits that are formulated by users, following a “reactive” approach to new product design. So far, proactive approaches to new product design that generate ex novo concepts for new varieties through user-led methods are rare. For example, consumer research can link culinary practices and cultural values to desirable future sensory properties of crops (94). This area is ready for data-driven approaches. Digital ethnographers have developed approaches to map microcultures that shape how consumers perceive new products (95).

A second limitation of current strategies is that they generally focus on defining trait preferences for the introduction of a single new variety. In reality, a variety is often part of a portfolio of varieties with complementary traits and functions in rural livelihoods. Farmers combine different types of varieties to address different crop production issues, to manage risks, and to serve for different end uses (96, 97). Rather than focusing on replacing a single variety, genebanks and breeding programs could attempt to provide a “menu” of different complementary varieties that farmers, processors, and consumers use within their cropping and livelihood systems. Also, variety replacement strategies could focus on existing varieties and their functions that are under threat from environmental change (98). A data-driven analysis of these interactions or complementary uses of varieties is important to understand the social implications of crop breeding decisions and to make effective use of existing crop diversity. Data-driven approaches to understand the complementary roles of varieties in cropping systems and livelihoods form an important new area for future research.

Innovation in the area of gender and socioeconomic differentiation holds the promise of increasing social benefits and gender equality outcomes of investments in public breeding programs and will be instrumental in minimizing unintentional negative consequences. Methodologically, this requires a careful design of quantitative strategies, which need to be informed by ethnographic, qualitative insights on aspects such as cultural gender norms and context-specific crop uses. Second, like in envirotyping (see above), the unit of analysis needs to be as granular as possible (the individual rather than the household or wider group) and needs to consider the tasks and level of control and engagement in different stages of crop use. Thirdly, more effort is needed to convert insights into gender and socioeconomic differentiation into decisions for crop improvement and diversity management, for example, through the development of social investment cases supported by empirical evidence. Recent studies have already given an indication toward the potential of data-driven strategies but need further integration to inform decision-making.

## Accountability and Breeding Progress Metrics

Crop breeding programs should take advantage of accelerated technological change, including in data-driven approaches, to make good use of available resources (1). Public and social investors in breeding need information on the social return on investment of breeding programs to prospect possible investment cases and to monitor performance of their investment portfolio. This means that breeding progress needs to be quantified, but there is wide agreement that metrics cannot just be output-oriented, such as the number of varieties released, or seed sales, which are indicators that are perhaps relevant for private profit, but not appropriate to quantify public benefit (99, 100).

Efficiency is an obvious lens through which to look at this, although researchers need to understand how this can be measured. The rate of genetic gain looks at the change in one or more target traits over time (1). This can also be assessed ex ante with the breeder’s equation, making it an attractive way to assess whether breeding programs make the right operational decisions to achieve high selection efficiency. This is a metric for technical efficiency. A broader concept is plant breeding efficiency, which also includes adoption—it has been proposed to measure it as the cost-benefit ratio, measuring benefit as the overall yield increase (100). However, this ignores other benefits, such as a reduction in production costs or pesticide use, increased nutrient content, or a contribution to gender and social equality. Breeding programs and investors will need different metrics to link breeding progress fully to the complex, representative contexts in which breeding products will be used (Fig. 1).

One way forward would be to create a composite index that incorporates different traits and their relative weights, based on socioeconomic data (101). This is a relatively resource-intensive strategy but could make the need for crop diversity more visible. Creating breeding indices will reveal possible negative trade-offs or correlations between different traits and divergent needs of different groups of stakeholders. Breeding programs can then decide in a data-driven way to address different needs by creating one or more breeding products.

To make complexity manageable, it has been suggested that breeding programs focus their efforts on creating breeding products that outperform a well-chosen, representative check variety or market leader (1). Performance testing could be operationalized by having stakeholders rank breeding products according to their overall appreciation, considering all relevant traits (80). This embraces the complexity of a comparison along many aspects, yet avoids the creation of a complex index. The associated metric in terms of breeding progress is reliability, the probability of outperforming a check (102). There are two limitations to this strategy. First, it will be difficult to assess trade-offs between the reliability of varieties according to different groups of stakeholders, for example, farmers vs. consumers. Second, this strategy focuses only on incremental change for an existing product category for which check varieties are available, not on breeding products that constitute an entirely new category.

Another type of analysis is needed to determine the progress of a breeding program across its entire portfolio. At this higher level of complexity, the trade-offs between different crop product users become even more salient. This is not just a question of assigning relative weights to different traits, but a matter of social choices that affect the relative benefit derived by groups of stakeholders—for example, farmers, processors, or consumers, men or women, who may have divergent interests. Making such choices is ideally done through a deliberative process that involves diverse perspectives from stakeholders. Such a deliberative process benefits more from a flexible data-driven approach than from a strongly model-driven approach.

## Toward Data-Driven Crop Diversity Management

In this perspective article, we have sketched how different aspects of crop diversity management can benefit from a data-driven approach. Many opportunities are emerging from new data-driven collaborations between disciplines. Data-driven approaches may help to surmount boundaries where other approaches have failed, for two reasons.

First, data-driven approaches that cross-disciplinary and organizational boundaries can bring greater accountability. Crop diversity management organizations that generate their own data on progress and impact can be perceived as grading their own exam. Independent data quality controls can ensure that data are trusted and inform important decisions on crop diversity management investments. Data-driven partnerships for social impact can ensure that independent verification of the added value of breeding products takes place, that future users are represented in the decision process, and that breeding programs respond to the criteria of breeding product users. Such new configurations can also allow bolder approaches in data-driven market research and diversifying product portfolios. Second, we think that data-driven approaches can be more successful than other integration pathways because they can deliver new insights from relatively incommensurable data and with a lower entry threshold to start collaborating between different disciplines. Relatively quick, tangible results can motivate collaborators to refine the approach by further developing conceptual models, harmonizing data collection, and generating tighter integration.

Alternative routes to integration are generally more demanding and have not been able to break the historical barriers discussed above. For example, integration around experiments involves factorial designs, which increase costs and complexity quickly, and are usually feasible for only a few additional factors. Integration around process-based crop models demands specific data and careful calibration, usually done for not more than a handful of genotypes. An important effect of these limitations is that usually, it is more difficult to work with a broad range of crop diversity or its interaction with environmental and socioeconomic heterogeneity. As a result, these methods used in isolation can inadvertently work as an excessively narrow funnel for crop diversity.

Experimental and modeling methods remain obviously important, but they can be combined with a broader range of data and machine learning methods in a data-driven approach. Flexible combinations of data and methods can lead to more insights with a lower upfront investment. Data-driven approaches do not necessarily imply that more data are needed for all aspects. Increased data volumes in one dimension can “lend” statistical power to another dimension (1), transfer learning can make it possible to train new machine learning models with less data (103), and more “permissive” data synthesis methods make it possible to combine heterogeneous datasets into larger datasets (81). Progress in machine learning and computing gives researchers access to a broad array of inductive, predictive analytics that allow for creative ways of linking datasets (104). Given these additional ways to extract value from data and accommodate complexity, a data-driven approach can generally deal with a broader range of crop diversity and interactions.

This capacity to handle more diversity throughout the crop diversity management system needs to be harnessed, not only to accelerate the development of crop breeding products but also to deliver a more diverse range of varieties into the hands of farmers and other crop diversity users. Summing up, we envisage an integrated crop diversity management system, in which data-driven approaches facilitate a more open innovation process. This process needs solid collaboration inside and outside research institutes and information-rich feedback between different disciplines and stakeholders. As data streams become richer and collaborations more meaningful, they allow the entire crop diversity management system to carry more information. Only by embracing the heterogeneity in environments and socioeconomic contexts can crop diversity management address the current challenges of climate change and rapid changes in consumer trends in the Global South, while supporting gender and social equality.

The implementation of the data-driven approach in crop diversity management system requires important changes in management strategies, policies, and investments. As breeding becomes a more interdisciplinary effort, decision-making processes need to be clearly structured in such a way that different perspectives are considered in breeding product advancement and different information flows come together at the right time, in the right format. This requires a substantial investment not only in data science capacity but also in information design, a distinct area of expertise that is still often underappreciated (105–107). The leadership of genebanks and breeding programs needs to be in the hands of professionals who are adept at interdisciplinary collaboration with an orientation to the end-users of crop diversity and have a good understanding of data science. The next generation of crop diversity professionals not only needs to have the skills to be able to work in interdisciplinary teams but also needs an excellent grasp of data science principles and scientific programming skills, beyond the ability to work with current tools and methods, which will soon be outdated. To build a strong data culture, policies that foster data sharing need to be in place.

Investors need to be aware that crop diversity management in the Global South cannot simply aim to close the supposed “gap” in comparison with the Global North. We have indicated important differences in capacities, regulations, and needs that require specific approaches. Policy makers and investors should contribute to carefully designing institutional structures and investments to prevent these efforts from remaining fragmented or being tied to certain crops only. In this way, a broad capacity should emerge to transform the crop diversity management system so that it uses data to benefit farmers, consumers, and other crop users.

## Data Availability

There are no data underlying this work.
